# Tc-99m radio-guided completion thyroidectomy for differentiated thyroid carcinoma

**DOI:** 10.4103/0972-3919.63593

**Published:** 2010

**Authors:** Savaş Karyağar, Oğuzhan Karatepe, Ömer Bender, Mehmet Mulazımoğlu, Tevfik Özpaçaci, Ercan Uyanık, Sevda S Karyağar, Orhan Yalçın, Yaşar Özdenkaya

**Affiliations:** Department of Nuclear Medicine, Istanbul, T.C.S.B. Okmeydani Training and Research Hospital, Turkey; 1 Department of General Surgery, Istanbul, T.C.S.B. Okmeydani Training and Research Hospital, Turkey

**Keywords:** Gamma probe, Tc-99m completion thyroidectomy, thyroid carcinoma

## Abstract

**Background:**

The purpose of this study is to investigate whether or not radio-guided surgery has any beneficial effects on completion thyroidectomy (CT) and the associated complication rates.

**Patients and Methods:**

Twenty-seven patients were scheduled for CT, for thyroid carcinoma, from December 2004 to June 2005, and were included in the study. All the patients had had initial thyroid surgery in other centers and been referred to our clinic for CT. Operation findings and the effectiveness of Tc-99m radio-guided CT were analyzed.

**Results:**

The intraoperative mean ratio of thyroid activity to background activity counted with a gamma probe was 1.3 ± 0.3. Average operation timing was 74 ± 9 minutes. Postoperatively, no residual tissue was detected in any of the patients with ultrasonography and thyroid scintigraphy. In the first postoperative month, serum TSH level was 61 ± 16.4 mIU / L, when preoperatively it was 7.3 ± 3.1 mIU / L (*P* < 0.001). In the postoperative period, one patient experienced temporary hypoparathyroidism (3.9%). Permanent hypoparathyroidism or recurrent laryngeal nerve damage was not detected in any patient.

**Conclusion:**

Tc-99 radio-guided CT is a reliable surgical method, which provides the detection and removal of residual thyroid tissues with minimal complications.

## INTRODUCTION

In differentiated thyroid carcinoma (DTC), the basic medical treatment is surgery.[1‐3] A considerable number of patients, undergoing subtotal resection as an initial surgical treatment, need reoperation for incidentally found thyroid carcinoma (TC), in a pathological examination. In patients with limited surgical resection, except for microcarcinoma and low risk, if residual tissue is more than 2 gms, then completion thyroidectomy in contemplated.[4]

Recurrence is more common in patients undergoing any procedure less than total or near total thyroidectomy. Fifty percent of all patients who develop local recurrence in the thyroid bed will eventually die of thyroid cancer.[5,6]

After the first operation, DTC patients with more than 2 g of remaining thyroid tissue or a radioactive I-131 uptake amount of more than 5%, should have a CT operation because of probable residual tissue tumoral focus resection, treatment with radioactive iodine (RAİ) and effective follow up to provide optimal outcome with throglobulin (Tg) estimation, to prolong a healthier life, to reduce locoregional and distant metastasis rate, and to rid the effects of RAİ treatments on widely residual thyroid, depending on the acute effects.[4,7‐10]

In inexperienced institutes, CT operations to get rid of residual thyroid tissues are not totally possible, because of the anatomic structure disorder, depending on the scars of the first operation, fibrosis results, and the ratio of parathyroid glands, with recurrent nerve disease depending on permanent complications, are increased.[11‐13] The intraoperative gamma probe is also used to detect and dissect lymph node recurrences and residual thyroid tissues.[14,15] In this study, the effectiveness of Tc-99m radio-guided surgery with a gamma probe, for detection and removal of the residual thyroid tissues, is examined. The aim of this study is to investigate whether or not radio-guided surgery has any beneficial effects on completion thyroidectomy (CT) and the associated complication rates.

## PATIENTS AND METHODS

### Patients

Twenty-seven patients, who had undergone thyroid surgery for goiter and then been diagnosed as DTC at histopathological examinations, were included in this present study, between December 2004 and 2005 June. The patients diagnosed with benign multinodular or solitary nodular goiter were operated in different clinics and in their pathology, DTC was detected. Subsequently, the patients were sent to our clinic. During the first operation, 23 patients who were already treated with bilateral subtotal thyroidectomy and patients who were treated with lobectomy and isthmectomy applied to our clinic with this information. Twenty-two patients were diagnosed with papillary TC histopathologically and five patients with follicular TC. All the patients' tumor diameters were ≥ 1 cm (centimeter).

In the operative period, physical examination thyroid scintigraphy (TS), thyroid and neck ultrasonography (USG), serum concentrations of TSH, Tg, anti thyroglobuline (Anti Tg), and an indirect laryngoscopic examination were performed. Six weeks after the first operation, the patients whose TSH level was < 30 mIU/ L, when thyroid hormone replacement was not taken, whose residual thyroid tissue was ≥ 2 g according to the USG and / or the USG results of the residual tissue, which had a suspicious discovery of malignancy, were diagnosed with CT indication.

### Gamma probe

Intraoperative monitoring was performed with the Navigator GPS System gamma probe using the superficial head and neck probe (C-Trak System; Care Wise, Morgan Hill, California). This system is a semiconductor probe based on a CdTe detector, with a head diameter of 10 mm, which provides feasible intraoperative handling. On the operation day, 10 min before the incision 0.5 ml 5 mCi Tc-99m pertechnetate was IV injected to the forearm. The counting ratio taken from the shoulder across the injected arm to the forearm by using the gamma probe was considered as background counting (BG). Both the thyroid beds were counted in three equal parts as upper, middle, and lower, in 10 second durations by using the gamma probe. The thyroid bed count and preoperative TS, revealing the remnant thyroid tissue, were compared. Another incision was performed at the former point of incision, and then them residual tissues in both thyroid beds were established and removed by using the gamma probe. After the removal of all these tissues, the thyroid bed was controlled for abnormal count with the gamma probe. Ratios were obtained by dividing the counting rate of the background activity before exicision (T / BG). Ratios after excision were measured by dividing the counting rates of the thyroid bed by the counting rates of the background activity (Tbed / BG). Completeness of the exicision is reflected by the low ratio of counting rates after excision.

Postoperative first day thyroid scintigraphy and serum calcium level dimension, sixth week serum fT4, fT3, TSH level, and USG were performed in all the patients.

The local Ethics Committee of our hospital approved the study and informed consent was obtained from all the patients participating in the trial.

### Statistical analyses

Data were analyzed using SPSS 15.0 for Windows. Results were expressed as mean ± SD. Wilcoxon t and Student t test were used for statistical analysis and *P* < 0.05 was accepted as significant.

## RESULTS

### Preoperative evaluation

**Figure 1:**
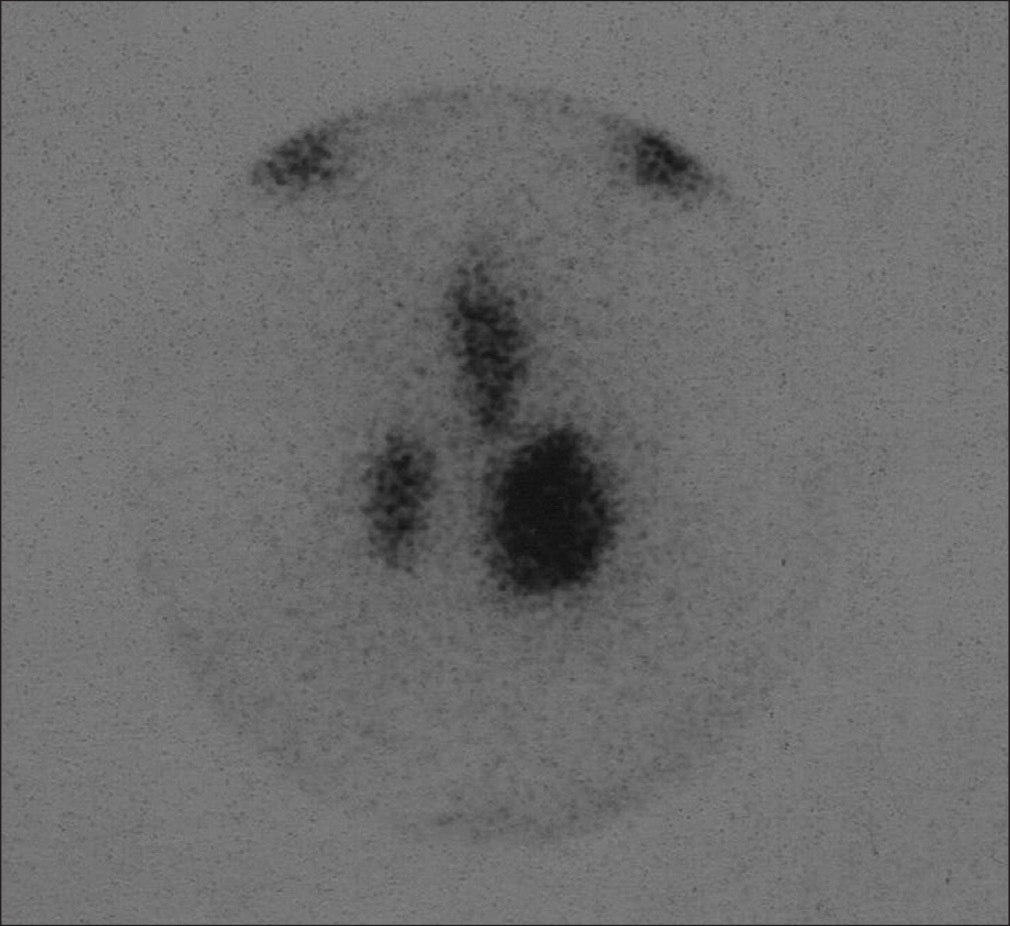
Tc-99m thyroid scintigraphy (before the completion thyroidectomy). There exists 3 distinct activity foci compatible with residual thyroid tissues in the right thyroid lobe, left thyroid lobe and pyramidal lobe

The mean age of the patients was 40 ± 14. The male / female ratio was 2: 25. The time between the first operation and CT was 92 ± 21 days. Patients preoperative mean TSH level was 7.3 ± 3.1 mIU / L (normal range 0.34 – 5.6 mIU / L). In all the patients, the evidence of remnant tissue was obtained preoperatively, both on TS and USG. In all the patients, including the patients on whom lobectomy was performed in the first operation, the bilateral remnant tissues were detected by TS and USG. In all the patients the remnant tissue average was 7.5 ± 1.1 g (range 5.5 – 9.7 g.). However, in seven patients, the nodule was detected on the remnant tissue [Figure 1]. During the physical and USG examination, lymphadenopathy was detected in one patient. Recurrent nerve injury was not found in any patient, but hypocalcemia was found in one patient.

**Figure 2:**
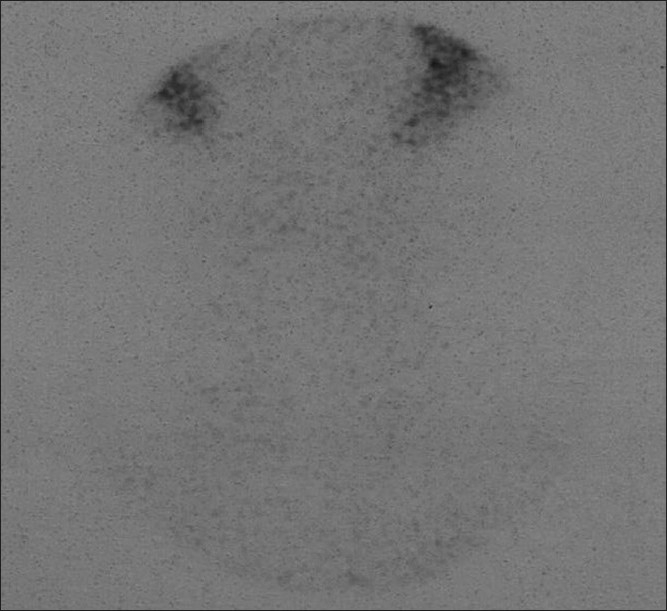
Tc-99m thyroid scintigraphy (after the completion thyroidectomy). No residual thyroid tissue was detected

Bilateral residual tissue excision was performed in 27 patients and modified neck dissection was performed in one patient. Remnant tissues detected by gamma probe in 25 patients were excised, while in two patients, on the berry ligament and cricothyroid cartilage region minimal remnant tissue was left because it was not causing recurrent nerve damage, and residual thyroid tissue excision was performed [Figure 2]. The intraoperative mean ratio of thyroid activity to background activity (T / BG) was detected as 9.3 ± 2.3 and the mean ratio of the thyroid bed activity to background activity, after excision (TB / BG) was 1.3 ± 0.2, (*P* < 0.001). The mean duration of the operation was 74 ± 9 min.

### The evaluation of the complications

In one patient temporary hypocalcemia was seen by gamma probe, after CT was performed, while permanent hypoparathyroidism was not seen in any patient. Transient or permanent recurrent laryngeal nerve damage was not seen in any patient.

### Histopatological evaluation

On pathological examination, the opposite lobe of four patients and the same lobe of one patient was with primer tumor. The tumor in these five patients (18.5%) was detected on residual tissue. On examination of nine patients (33.3%) in the first operation and CT together, multicentric tumor existence was detected.

On the first postoperative day, on TS, and in the fourth week on USG, existence of remnant tissues was not seen in 25 patients, and minimal residual thyroid tissue existence was seen in two patients. In the sixth week after the operation, the TSH value was > 30 mIU/L in all the patients, while the mean TSH was 61 ± 16.4 mIU/L (*P* < 0.001).

## DISCUSSION

In TC patients, the presence of residual thyroid tissue following initial surgery has major implications for post thyroidectomy treatment and testing. For the RAI ablation therapy to be effective, the preferred TSH level should be higher than 30 mIU / L. Reoperation should be performed in patients with incidentally-found TC, if the histological criteria mandates RAI ablation and there is a large volume of thyroid remnant.

Recurrence is more common in patients who undergo any procedure less than total or near-total thyroidectomy. Fifty percent of all the patients who have developed local recurrence in the thyroid bed finally die due to TC.[5,6]

Multifocal papillary TC has been shown to be present in 25 to 88% of the patients with papillary TC and after subtotal thyroidectomies, the residual carcinoma risk is 22 – 64%.[16‐19] In the present study, after CT, residual carcinoma was detected in five (18.5%) patients. In the present study, the cause of low-rate residual carcinoma was because the excision of the existing nodules, in the first operation performed surgically, was thought to be effective. TC is diagnosed very often in our country through pathological examinations, in which most patients are diagnosed with having preoperative benign illness and treated by single direction lobectomy or bilateral subtotal thyroidectomy. In this study, all the patients (23 of whom had undergone bilateral subtotal thyroidectomy, and four of whom had undergone lobectomy and istmectomy) underwent initial surgery for benign multinodular or solitary nodular goiter. Twenty-seven patients had applied to our clinic. Histopathologically, 22 patients were diagnosed with papillary TC and five patients with follicular TC. The diameter of the tumor in all the patients was ≥ 1 cm. In all the patients, bilateral residual thyroid tissue was detected by preoperative USG and TS and every patient's total remnant tissue was ≥ 2 g, and in one the patient remnant thyroid tissue was at least 1 g and TSH was ≥ 30 mIU/L.

After the first operation, the DTC patients who had more than 2 g of remnant tissue or a radioactive I-131 uptake amount of more than 5% had to have a CT operation because of probable tumoral focus resection on the residual tissue, as also effective follow-up and treatment with RAI, to provide an optimal follow-up with the level of postoperative tg., to prolong life without metastases and to remove the effects of RAI treatments on the widely residual thyroid tissue, depending on the acute effects.[4,7‐10]

The hospitals performing completion thyroidectomy show complication rate similar to total thyroidectomy. However, the previous thyroid surgery cause extensive scarring which makes re-do surgery technically difficult and leads to complication related to parathyroid and recurrent laryngeal nerve. In literature, according to CT operations, 2 – 5% recurrent laryngeal nerve damage, 8 – 15% temporary, and 0 – 3,5% permanent hypoparathyroidism have been reported.[6,7,11‐13,20,21]

In DTC patients, surgery with gamma probe on sentinel node applications, locoregional residue, and recurrent and metastatic lymph node surgical treatments have been used in recent years.[15,22‐25] Boz *et al*. and Rubello *et al*. proposed radio-guided surgery for the intraoperative localization and resection of locoregional, non-functioning (I-131-negative) DTC recurrences with Tc-99m MIBI.[14,22,25] Rubello *et al*. reported a lesion to background ratio higher than 2.0 in all cases (mean ± SD = 3.2 ± 0.8) at operation. CT with gamma probe-guided surgery was first described by Erbil *et al*. In their study, thyroid activity to background activity (Tbed / BG) was 1.3 ± 0.3.[26]

In the Tc-99m radio-guided surgery, the thyroid bed was counted with the gamma probe and areas with a higher count than the background activities were explored. The maximum uptake by the thyroid gland occurred between 20 and 30 min. The thyroid bed counting was performed approximately 10 – 20 min after the injection of Tc-99m. The median duration of the surgery was 74 ± 9 min, which made Tc-99m an appropriate agent to use. After removal of the residual thyroid tissue, the thyroid bed was re-accounted to check for the presence of abnormal counts. The discrimination of fibrous non-thyroid tissues was also carried out by the intraoperative use of the gamma probe. Preoperative gamma countings were compared with the target thyroid tissue and when thyroid loj counting and background counting were equal, the operation was stopped. The intraoperative mean ratio of thyroid activity to background activity was higher than the ratio found in Erbil *et al*'s study.

The gamma probe permitted us to look for neoplastic foci with high sensitivity and specificity and to remove the metastatic lymph nodes resistant to radioiodine therapy. It allowed for the removal of pathological areas of uptake in the neck in most patients; moreover, it allowed for the detection of some difficult-to-identify additional tumoral foci inside the areas of the sclerosis or behind vascular structures, which were not seen during pre-surgical evaluation. In confirmation of these results, in most cases, the surgeons expressed a positive opinion about the procedure, describing it as decisive or favorable. In spite of the disappearance of the anatomic plans, because of scar and fibrosis that was caused by the use of the gamma probe on the operation area, in the first operation, the thyroid tissue could be distinguished from the surrounding area. Therefore, the structure that did not have thyroid tissues would not be excised unnecessarily; parathyroid and recurrent nerve protection could be provided by relatively reaching the anatomic plans, for determining the thyroid tissues accurately, with a gamma probe.

In this study, preoperative mean TSH was 7.3 ± 3.1 mIU / L in the patients on whom CT was performed by using the gamma probe, while in the postoperative period it was 61 ± 16.4 mIU / L, (p < 0.001). In all the patients postoperative TSH was > 30 mIU / L. In postoperative controls, no residual tissue was detected in TS or USG of 25 patients out of 27, while the minimal focal focus condition in the residual tissue was found in two patients. This finding indicated that during the operation, Tc-99m radio-guided surgery by using gamma probe and CT could be performed with minimal complications, despite the scar and fibrosis thyroid tissues.

In conclusion, in inexperienced surgical clinics where CT is performed, Tc-99m radio-guided CT by using gamma probe will be a reliable method that improves the surgical learning curves for DTC patients.
